# Clinical Significance of Peripheral Arterial Disease Evaluation in Patients with Antineutrophil Cytoplasmic Antibody-Associated Vasculitis

**DOI:** 10.3390/medicina61061074

**Published:** 2025-06-11

**Authors:** Jeong Yeop Whang, Lucy Eunju Lee, Jang Woo Ha, Oh Chan Kwon, Yong-Beom Park, Sang-Won Lee

**Affiliations:** 1 Department of Medicine, Yonsei University College of Medicine, Seoul 03722, Republic of Korea; jywhang03@naver.com; 2 Division of Rheumatology, Department of Internal Medicine, Yonsei University College of Medicine, Seoul 03722, Republic of Korea; elee@yuhs.ac (L.E.L.);; 3 Division of Rheumatology, Department of Internal Medicine, Yongin Severance Hospital, Yonsei University College of Medicine, Yongin 16995, Gyeonggi-do, Republic of Korea; 4 Division of Rheumatology, Department of Internal Medicine, Gangnam Severance Hospital, Yonsei University College of Medicine, Seoul 06273, Republic of Korea; 5 Institute for Immunology and Immunological Diseases, Yonsei University College of Medicine, Seoul 03722, Republic of Korea

**Keywords:** antineutrophil cytoplasmic antibody, vasculitis, peripheral arterial disease, test, kidney

## Abstract

*Background and Objectives*: This study investigated the frequency and clinical significance of subclinical but substantial peripheral arterial disease (PAD), identified using PAD evaluation, including pulse volume recording/ankle–brachial index (PVR/ABI), transcutaneous oxygen pressure (TcpO2), and skin perfusion pressure (SPP) tests in patients with antineutrophil cytoplasmic antibody-associated vasculitis (AAV). *Materials and Methods:* This study included 54 patients with PAD evaluation results at or after AAV diagnosis. PVR/ABI and/or TcpO2 and/or SPP were performed on the same day. Abnormal PVR/ABI, TcpO2, and SPP were defined as PVR/ABI < 0.97, TcpO2 < 40 mmHg, and SPP < 50 mmHg, respectively. Poor outcomes included all-cause mortality, end-stage kidney disease (ESKD), cerebrovascular accidents, and acute coronary syndrome after PAD evaluation. *Results:* The median age of the 54 patients was 67 years, and 48.1% were male. In total, 3 of 54 patients (5.6%), 6 of 16 (37.5%), and 6 of 23 (26.1%) had abnormal PVR/ABI, TcpO2, and SPP, respectively. The concordance rate between abnormal PVR/ABI and abnormal TcpO2 or SPP was very low. Among the 54 patients, 5 (9.3%) died, and 2 (3.7%) progressed to ESKD. Abnormal SPP was significantly associated with cutaneous and renal manifestations at the time of PAD evaluation and had the potential to predict progression to ESKD during follow-up in patients with AAV. *Conclusions:* This study is the first to reveal the clinical usefulness of PAD evaluation: abnormal SPP may have the potential to identify subclinical but substantial PAD and can predict simultaneous kidney involvement as well as future progression to ESKD in patients with AAV.

## 1. Introduction

Antineutrophil cytoplasmic antibody (ANCA)-associated vasculitis (AAV) is an autoimmune systemic vasculitis characterised by fibrinoid necrotising vasculitis in small vessels including capillaries, arterioles, venules, and occasionally medium-sized arteries [1]. AAV has three subtypes according to subtype-specific unique and distinct clinical features: microscopic polyangiitis (MPA), granulomatosis with polyarteritis (GPA), and eosinophilic GPA (EGPA) [1,2,3]. AAV can affect almost all major organs, and the extent and severity of organ involvement have a critical impact on prognosis [4]. In real-world clinical practice, we occasionally (or very rarely) encounter AAV patients who complain of pain and circulatory disorders (e.g., Raynaud’s phenomenon) in the lower extremities, regardless of cutaneous symptoms [5,6,7]. Lower-extremity computed tomography (CT) angiography is currently and widely used to detect and identify peripheral arterial disease (PAD). However, it is very rare that clear arterial insufficiency is found on lower-extremity CT angiography unless severe and obvious arterial insufficiency leading to leg necrosis develops [8,9]. As such, the need for an accurate and non-invasive method to recognise and identify subclinical but substantial PAD in patients with AAV has increased. Recently, non-invasive tests such as pulse volume recording/ankle–brachial index (PVR/ABI), transcutaneous oxygen pressure (TcpO2), and skin perfusion pressure (SPP) tests have been performed to determine the presence of PAD, and their clinical efficacy has been verified [10,11,12,13,14,15]. Hence, in this study, we selected AAV patients with PAD evaluation results, including PVR/ABI and/or TcpO2 and/or SPP tests, from a cohort of AAV patients. We also investigated the frequency and clinical significance of subclinical but substantial PAD identified by PAD evaluation in AAV patients.

## 2. Materials and Methods

### 2.1. Patients

**Figure 1:**
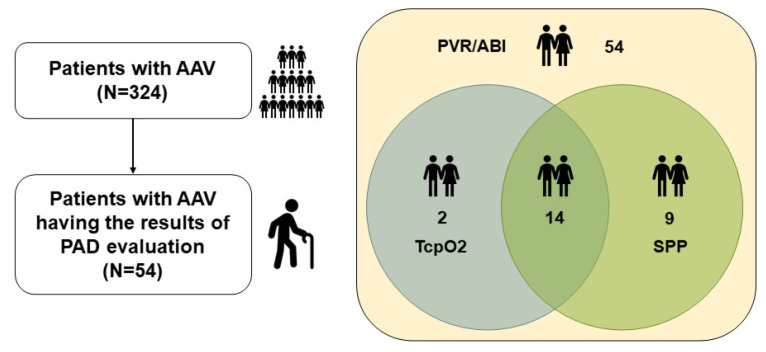
Patient selection. AAV: antineutrophil cytoplasmic antibody-associated vasculitis; PAD: peripheral arterial disease; PVR: pulse volume recording; ABI: ankle–brachial index; TcpO2: transcutaneous oxygen pressure; SPP: skin perfusion pressure.

Among the 324 patients with AAV enrolled in the Severance Hospital ANCA-associated Vasculitides (SHAVE) cohort, an observational cohort of AAV, we selected and included 54 patients with PAD evaluation results, such as PVR/ABI and/or TcpO2 and/or SPP tests, at/after AAV diagnosis. Their medical records were retrospectively reviewed. All patients fulfilled the revised 2012 Chapel Hill Consensus Conference Nomenclature of Vasculitides [1], the algorithm for AAV and polyarteritis nodosa proposed by the European Medicine Agency in 2007 [2], and the classification criteria for MPA, GPA, and EGPA proposed by a joint group of the American College of Rheumatology and the European Alliance of Associations for Rheumatology in 2022 (the 2022 ACR/EULAR criteria) [16,17,18,19]. All patients were diagnosed with AAV at our hospital and had well-documented medical records for collecting clinical data at the time of PAD evaluation. More detailed inclusion criteria for the SHAVE cohort have been described in our previous studies [20,21,22]. Of the 324 patients, 54 underwent PAD evaluation tests owing to subjective symptoms such as nonspecific leg pains and/or circulatory discomfort, and thus they were included in this study. All 54 patients had the results of PVR/ABI tests. Of the 54 patients, 16 and 23 had the results of TcpO2 and SPP tests, respectively, and further, 14 underwent both TcO2 and SPP tests (Figure 1). This study was approved by the Institutional Review Board (IRB) of Severance Hospital, Seoul, Republic of Korea on 10 November 2020 (IRB No. 4-2020-1071), and conducted according to the Declaration of Helsinki. Owing to the retrospective design of the study and the use of anonymised patient data, the requirement for written informed consent was waived.

### 2.2. Clinical Data at PAD Evaluation

The variables recorded at the time of PAD evaluation included demographic data (age, sex, body mass index, and smoking history), AAV subtype, ANCA type, and positivity, and AAV-specific indices including the Birmingham vasculitis activity score (BVAS), and the five-factor score (FFS) [23,24]. The results of routinely performed laboratory tests and acute-phase reactants at the time of PAD evaluation were also collected (Table 1). Perinuclear (P)-ANCA and cytoplasmic (C)-ANCA were detected using an indirect immunofluorescence assay, whereas myeloperoxidase (MPO)-ANCA and proteinase 3 (PR3)-ANCA were measured using an immunoassay [25]. According to the 2022 ACR/EULAR criteria for AAV [16,17,18], P- and C- ANCA alongside MPO-ANCA and PR3-ANCA were accepted as ANCA results. Specific clinical manifestations of each item of the BVAS are as follows: (i) general: myalgia, arthralgia/arthritis, fever, and weight loss > 2 kg; (ii) cutaneous: skin infarct, purpura, ulcer, gangrene, and other skin vasculitis; (iii) mucous/ocular: mouth ulcers, genital ulcers, adnexal inflammation, proptosis, scleritis/episcleritis, conjunctivitis/blepharitis/keratitis, blurred vision, sudden visual loss, uveitis, and retinal changes; (iv) otorhinolaryngological: nasal passage abnormalities, paranasal sinusitis, subglottic stenosis, and hearing loss; (v) pulmonary: wheeze, nodules or cavities, pleural effusion/pleurisy, infiltrate, endobronchial involvement, diffuse alveolar haemorrhage, and respiratory failure; (vi) cardiovascular: loss of pulse, valvular heart disease, pericarditis, ischaemic cardiac pain, cardiomyopathy, and congestive cardiac failure; (vii) gastrointestinal: peritonitis, bloody diarrhoea, and ischaemic abdominal pain; (viii) renal: hypertension, proteinuria, haematuria, and serum creatinine elevation; (ix) nervous systemic: headache, meningitis, organic confusion, seizures, cerebrovascular accident, spinal cord lesion, cranial nerve palsy, sensory peripheral neuropathy, and mononeuritis multiplex [23].

### 2.3. PAD Evaluation

The PAD evaluation included PVR/ABI, TcpO2, and SPP tests, which were conducted using the same methods as described in previous studies [10,12,15]. In cases where TcpO2 and SPP tests were performed, they were completed alongside PVR/ABI tests on the same day. Values measured in both the right and left legs were collected, and when either fell into an abnormal range, it was considered an abnormal value. Based on the normal ranges of PAD evaluation values in our hospital, abnormal PVR/ABI, TcpO2, and SPP were defined as PVR/ABI < 0.97, TcpO2 < 40 mmHg, and SPP < 50 mmHg, respectively [15] (Table 1). The time gap from AAV diagnosis to PAD evaluation was calculated (Supplementary Figure S1).

### 2.4. Poor Outcomes During Follow-Up

Data regarding all-cause mortality (ACM), end-stage kidney disease (ESKD), cerebrovascular accident (CVA), and acute coronary syndrome (ACS) after PAD evaluation were collected as poor outcomes of AAV during follow-up [26,27,28]. The follow-up duration based on each poor outcome after PAD evaluation was defined as the period from the PAD evaluation to each poor outcome occurrence for patients with each poor outcome, whereas it was defined as the time to the last visit for those without poor outcomes (Supplementary Figure S1).

### 2.5. Statistical Analyses

All statistical analyses were performed using SPSS version 26 (IBM Corporation, Armonk, NY, USA) for Windows (Microsoft Corporation, Redmond, WA, USA). Continuous and categorical variables were expressed as medians (25 and 75 percentiles), and numbers (percentages). Significant differences between the two categorical variables were analysed using the chi-square and Fisher’s exact tests with Yates continuity correction. A comparison of the cumulative survival rates between the two groups was performed using Kaplan–Meier survival analysis with the log-rank test. The odds ratio (OR) was obtained using univariable logistic regression analysis. *p* < 0.05 was considered to be statistically significant.

## 3. Results

### 3.1. Characteristics of Patients with AAV at PAD Evaluation

The median age of the 54 patients was 67 years, and 48.1% were male.

Among the 54 patients, 12, 10, and 32 were diagnosed with MPA, GPA, and EGPA, respectively. MPO-ANCA (or P-ANCA) and PR3-ANCA (or C-ANCA) were detected in 41 and 5 patients. The median BVAS and FFS were 12.0 and 1.0. Also, the median erythrocyte sedimentation rate (ESR) and C-reactive protein (CRP) were 86.5 mm/h and 19.0 mg/L (Table 1).

### 3.2. Results of PAD Evaluation

The median right and left PVR/ABI, TcpO2, and SPP values were 1.19, 1.19, 49.0 mmHg, 48.0 mmHg, 56.0 mmHg, and 57.0 mmHg, respectively. A total of 3 of the 54 (5.6%) patients exhibited abnormal PVR/ABI. Additionally, 6 of the 16 (37.5%) patients with TcpO2 results showed abnormal TcpO2, and 6 of the 23 (26.1%) patients with SPP results had abnormal SPP as well. The median time gap between AAV diagnosis and PAD evaluation was 10 months (Table 1).

### 3.3. Concordance Rate of Abnormal PAD-Related Values Between PVR/ABI and TcpO2 or SPP

Among the 16 patients with both PVR/ABI and TcpO2 test results, none exhibited abnormal PVR/ABI and TcpO2 simultaneously, resulting in a concordance rate between abnormal PVR/ABI and TcpO2 of 0%. Conversely, 56.3% of the patients had normal PVR/ABI and TcpO2 tests. Additionally, among the 23 patients with both PVR/ABI and SPP results, only 1 patient exhibited abnormal PVR/ABI and SPP simultaneously, leading to a concordance rate between abnormal PVR/ABI and SPP of 4.4%. On the other hand, 65.2% of the patients had normal values in both the PVR/ABI and SPP tests (Table 2).

### 3.4. Systemic Manifestations and Poor Outcomes

At PAD evaluation, the most commonly observed systemic manifestation based on the items of the BVAS was pulmonary (66.7%), followed by renal (61.1%) and nervous systemic (51.9%) manifestations. During follow-up after PAD evaluation, of the 54 patients, 5 (9.3%) died and 2 (3.7%) progressed to ESKD. Also, CVA and ACS occurred in 10 (18.5%) and 4 (7.4%) patients (Table 3).

### 3.5. Comparison of Systemic Manifestations and Poor Outcomes According to Abnormal PAD Evaluation

In terms of systemic manifestations at PAD evaluation, patients with abnormal SPP exhibited cutaneous manifestation more frequently than those with normal SPP (83.3% vs. 5.9%, *p* = 0.001). In addition, renal manifestation was observed in patients with abnormal SPP more often than in those with normal SPP (100% vs. 41.2%, *p* = 0.012). Meanwhile, no significant differences in systemic manifestations between patients with abnormal PVR/ABI and TcpO2 and those with normal values were found. However, in terms of AAV poor outcomes during follow-up, there were no significant differences in the occurrence of AAV poor outcomes according to abnormal PVR/ABI, TcpO2, and SPP at PAD evaluation (Table 4).

### 3.6. Comparison of Cumulative ESKD-Free Survival Rates According to Abnormal SPP

**Figure 2:**
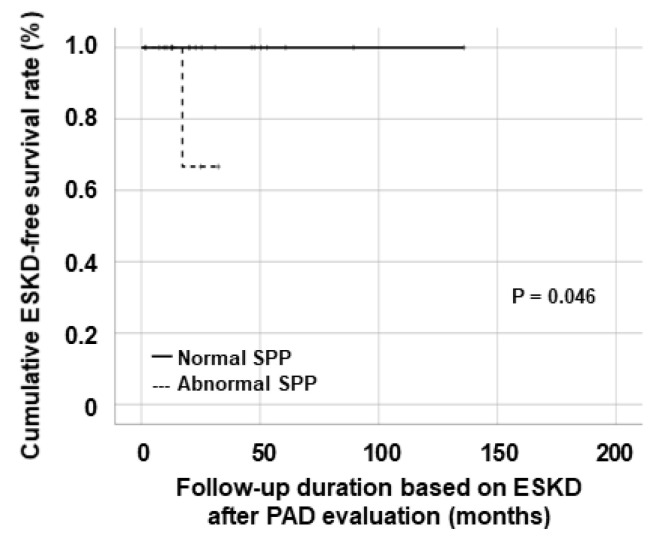
Comparison of the cumulative ESKD-free survival rates. ESKD: end-stage kidney disease; SPP: skin perfusion pressure.

Regarding the association of abnormal PAD-related values with future AAV poor outcomes, among the three methods for identifying subclinical but substantial PAD, only patients with abnormal SPP exhibited a significantly lower cumulative ESKD-free survival rate than those with normal SPP (*p* = 0.046) (Figure 2).

## 4. Discussion

In this study, we investigated the frequency of subclinical but substantial PAD based on PVR/ABI, TcpO2, and SPP tests in AAV patients and obtained several interesting findings [10,11,12,13,14,15]. Firstly, abnormal PVR/ABI, TcpO2, and SPP values were found in 3 of 54 (5.6%), 6 of 16 (37.5%), and 6 of 23 (26.1%) patients with AAV with PAD-related test results, respectively. Although the reliability is not high because only a small number of patients underwent relevant PAD-related tests, we cautiously suggest that the frequencies of subclinical but substantial PAD in AAV patients based on PVR/ABI, TcpO2, and SPP tests were 5.6%, 37.5%, and 26.1%, respectively. Secondly, the concordance rate between abnormal PVR/ABI and TcpO2 was 0%, whereas that between abnormal PVR/ABI and SPP was identified as 4.4%. Third, among three abnormal PAD-related values, only abnormal SPP was significantly associated with cutaneous and renal manifestations. However, none of them was associated with future poor outcomes of AAV. Fourth, nonetheless, in survival rate analyses, among three PAD-related values, only abnormal SPP was also significantly associated with future progression to ESKD in patients with AAV. Therefore, we conclude that TcpO2 or SPP tests might be more useful than PVR/ABI tests in detecting subclinical but substantial PAD in AAV. Also, we demonstrated that abnormal SPP was significantly associated with cutaneous and renal manifestations at PAD evaluation and had the predictive potential for progression to ESKD during follow-up in patients with AAV.

Roughly, PVR/ABI tests can be said to reflect PAD at the level of relatively medium-sized arteries, while TcpO2 and SPP tests can be said to estimate PAD at the level of relatively small-sized arterioles [12,15]. From the perspective of the general population, TcpO2 and SPP tests are not strongly recommended and are known to be of little help in cases where no ischemic ulcer or gangrene in the lower extremities occurs [15]. In addition, in cases where no significant arterial stenosis or occlusion is found on lower-extremity CT angiography, PVR/ABI tests are recommended to be performed first, rather than TcpO2 and/or SPP tests. And when the PVR/ABI value is 0.75 or higher (based on the upper limit of the normal range), additional tests such as TcpO2 and/or SPP tests are not strongly recommended clinically [29]. However, in cases where the PVR/ABI value is 0.74 or lower and accompanied by leg pain and circulatory disorders, regardless of cutaneous manifestations, TcpO2 and/or SPP tests may be further considered but are not mandatory [12,15]. However, from the perspective of patients with AAV, the clinical necessity of TcpO2 and SPP tests is by no means trivial. Given that AAV can provoke inflammation in capillaries with adjacent arterioles and venules, and further, medium-sized artery involvement [30], it can be speculated that not only PVR/ABI tests but also both TcpO2 and SPP tests may theoretically be useful in the early detection of subclinical but substantial PAD in patients with AAV. Nonetheless, to date, there has been no guideline to perform TcpO2 and SPP tests in patients with AAV in addition to PVR/ABI tests. Therefore, this study has a clinical advantage in that it is the first to suggest the clinical utility of TcpO2 and SPP tests for identifying (or suspecting) subclinical but substantial PAD in patients with AAV.

PAD has not been highlighted in AAV patients to date. First of all, among the items of the BVAS, the cardiovascular item has only a subitem that suggests PAD, ‘Loss of pulse’, which is defined as the clinical absence of peripheral arterial pulsation in any limb [23]. However, in real clinical practice, ‘Loss of pulse’ is extremely rare in most patients complaining of discomfort in the lower extremities. Therefore, the BVAS, which reflects AAV activity, seems to be of no interest in subclinical but substantial PAD. Additionally, the vasculitis damage index (VDI), which reflects the extent of damage caused by AAV, only mentions clinically significant arterial insufficiencies such as ‘Absence pulses in one limb’, ‘Major vessel stenosis’, and ‘Claudication > 3 months’ but does not describe subclinical but substantial PAD [31]. Hence, we have confronted three dilemmas. The first is whether higher frequencies of abnormal TcpO2 and SPP compared to that of abnormal PVR/ABI may directly indicate a higher frequency of subclinical but substantial PAD. Conversely, the second is whether patients with normal TcpO2 and SPP may be free from AAV-related or unrelated subclinical but substantial PAD. The third is by what method subclinical but substantial PAD suspected based on abnormal TcpO2 and SPP may be confirmed. Nevertheless, given that TcpO2 and SPP may reflect the potential of delayed wound healing due to ischemia leading to changes in the microvasculature and that arterial insufficiency caused by AAV may be difficult to detect by routine imaging studies, we believe that the proper treatment for subclinical but substantial PAD should actively be considered and initiated in patients with AAV who present with clinical symptoms of the lower extremities and have abnormal TcpO2 or SPP.

The interesting part of the results of this study is that abnormal SPP showed significant associations with cutaneous and renal manifestations at the same time point of PAD evaluation. While the link between skin lesions of the lower extremities and reduced SPP values was somewhat predictable, the association between kidney involvement and abnormal SPP values was not expected at all. It might be impossible to suggest the exact and direct mechanism of the association between abnormal SPP and renal manifestation of AAV. However, previous studies have discovered that certain clinical features of AAV are associated with renal involvement and that nail-fold capillary abnormalities were significantly associated with certain clinical features of AAV [32,33]. Therefore, given that the skin and kidneys are the organs where the smallest blood vessels, at the capillary level, are distributed and have the highest capillary density, it is reasonable to infer a correlation between abnormal SPP and renal manifestation, particularly glomerular capillaritis affected by AAV. In addition, the finding that abnormal SPP at PAD evaluation could predict future progression to ESKD further supports this inference because the initial alteration in renal function is one of the critical risk factors for ESKD in the general population. Therefore, this study has another clinical advantage in that it is the first to suggest the clinical utility of SPP tests for not only implying the possibility of renal involvement of AAV but further predicting future progression to ESKD in patients with AAV.

Meanwhile, the influence of atherosclerosis-related risk factors alongside AAV on abnormal SPP was also investigated using logistic regression analysis to minimise the confounding factors. The atherosclerosis-related risk factors were divided into three categories, general influence factors, AAV-specific influence factors, and inflammation-related influence factors, and applied to the statistical analysis [34,35,36]. Firstly, in terms of general influence factors, age (*p* = 0.550), sex (*p* = 0.901), BMI (*p* = 0.814), hypertension (*p* = 0.369), diabetes mellitus (*p* = 0.137), dyslipidaemia (*p* = 1.000), and serum creatinine (*p* = 0.322) were not significantly associated with abnormal SPP. Secondly, in terms of AAV-specific influence factors, the BVAS (*p* = 0.678), the FFS (*p* = 0.162), MPO-ANCA (or P-ANCA) positivity (*p* = 0.858), and PR3-ANCA (or C-ANCA) positivity (*p* = 0.999) were not significantly associated with abnormal SPP either. Lastly, in terms of inflammation-related influence factors, erythrocyte sedimentation rate (*p* = 0.282), C-reactive protein (*p* = 0.138), haemoglobin (*p* = 0.403), platelet count (*p* = 0.650), and serum albumin (*p* = 0.552) were not significantly associated with abnormal SPP at all. Therefore, we concluded that abnormal SPP was not significantly affected by several confounding atherosclerosis-related risk factors. However, because the number of patients included in this study was insufficient, the possibility of errors that could be interpreted as exaggerated results cannot be ruled out.

On the other hand, given that metabolic syndrome, including hypertension, type 2 diabetes mellitus, and dyslipidaemia, may have a critical influence on atherosclerosis occurrence in the main vessels [37,38], we investigated the effects of such comorbidities on the cross-sectional results of PAD evaluation tests. Using the chi-square and Fisher’s exact tests with Yates continuity correction, we compared the abnormal results of PAD evaluation tests according to the presence of each comorbidity; however, we found no significant differences between the two groups.

This study has an advantage in that it is the first to report the frequency of subclinical but substantial PAD in AAV patients with leg pain and circulatory disorders, regardless of cutaneous manifestations using the results of RVR/ABI and/or TpcO2 and/or SPP tests. Furthermore, this study also has another advantage in that this is the first to suggest the possible association of abnormal SPP with renal manifestations as well as future progression to ESKD. However, this study has several limitations. The critical limitation is the small number of AAV patients with PAD evaluation results, meaning the results of this study cannot be generalized and applied to all patients with AAV. The retrospective study design is another limitation, resulting in not controlling the confounding factors affecting PVR/ABI, TcpO2, and SPP values, such as serious ischaemic gangrenes or necrosis and severe oedema in the lower extremities. In particular, despite the clinical usefulness of ultrasound on the vessels of the lower extremities, there were no patients who underwent ultrasonography on the vessels of the lower extremities in the present study. This is because the performance of ultrasound tests for evaluating PAD in the lower extremities was not included in the protocol of the cohort of AAV patients in this institute. If there had been the results of ultrasound tests on the main vessels of the lower extremities, they could have helped to verify as well as increase the robustness of the results of this study on the clinical utility of PAD evaluation in patients with AAV. Also, because not all patients complaining of nonspecific leg pains and/or circulatory discomfort underwent PAD evaluation tests, a substantial selection bias on the underestimated incidence of abnormal PAD evaluation results in this study cannot be excluded. In addition, the limitations that need to be improved are the inability to observe the alteration pattern through continuously measured PAD-related values and the very low incidence rate of poor outcomes during follow-up after PAD evaluation. We believe that a future study with more patients and serially measured PAD-related values will provide more reliable and dynamic information on the clinical significance of PVR/ABI, TcpO2, and SPP tests in patients with AAV.

## 5. Conclusions

This study is the first to reveal the clinical usefulness of PAD evaluation: specifically, abnormal SPP may have the potential to identify subclinical but substantial PAD and can predict simultaneous kidney involvement, as well as future progression to ESKD, in patients with AAV.

## Figures and Tables

**Table 1:** Characteristics of patients with AAV at PAD evaluation (*n* = 54).

Variables Regarding Clinical Data	Value
Demographic data	
Age (years)	67.0 (56.0–71.0)
Male sex (*n*, (%))	26 (48.1)
Female sex (*n*, (%))	28 (51.9)
BMI (kg/m^2^)	22.9 (21.4–24.9)
Ex-smoker (*n*, (%))	3 (5.6)
AAV subtype (*n*, (%))	
MPA	12 (22.2)
GPA	10 (18.5)
EGPA	32 (59.3)
ANCA type and positivity (*n*, (%))	
MPO-ANCA (or P-ANCA) positivity	41 (75.9)
PR3-ANCA (or C-ANCA) positivity	5 (9.3)
Both ANCA positivity	1 (1.9)
ANCA negativity	9 (16.7)
AAV-specific indices	
BVAS	12.0 (5.8–19.0)
FFS	1.0 (0–3.0)
Laboratory results	
White blood cell count (/mm^3^)	9750.0 (6877.5–13,140.0)
Haemoglobin (g/dL)	11.5 (10.0–12.7)
Platelet count (×1000/mm^3^)	329.0 (240.5–396.3)
Fasting glucose (mg/dL)	101.0 (88.0–114.5)
Blood urea nitrogen (mg/dL)	16.7 (12.3–23.2)
Serum creatinine (mg/dL)	0.8 (0.7–1.1)
Total cholesterol (mg/dL)	164.0 (130.8–194.0)
Serum total protein (g/dL)	6.8 (6.1–7.2)
Serum albumin (g/dL)	3.6 (3.1–4.1)
Complement 3 (mg/dL)	122.4 (103.0–134.3))
Complement 4 (mg/dL)	25.3 (20.2–31.2)
Acute-phase reactants	
ESR (mm/hr)	86.5 (23.8–120.0)
CRP (mg/L)	19.0 (1.8–105.1)
Comorbidities (*n*, (%))	
Hypertension	16 (29.6)
Type 2 diabetes mellitus	17 (31.5)
Dyslipidaemia	9 (16.7)
Results of PAD evaluation	
Continuous variables	
PVR/ABI right (*n* = 54)	1.19 (1.10–1.25)
PVR/ABI left (*n* = 54)	1.19 (1.11–1.26)
TcpO2 right (mmHg) (*n* = 16)	49.0 (38.3–58.3)
TcpO2 left (mmHg) (*n* = 16)	48.0 (33.8–51.0)
SPP right (mmHg) (*n* = 23)	56.0 (50.0–68.0)
SPP left (mmHg) (*n* = 23)	57.0 (50.0–66.0)
Abnormal results ((*n*, (%))	
Abnormal any PVR/ABI (*n* = 54)	3 (5.6)
Abnormal any TcpO2 (*n* = 16)	6 (37.5)
Abnormal any SPP (*n* = 23)	6 (26.1)
Time gap between AAV diagnosis and PAD evaluation (months)	9.99 (0–65.9)

Values are expressed as the median (25th–75th percentile) or *n* (%). AAV: ANCA-associated vasculitis; ANCA: antineutrophil cytoplasmic antibody; PAD: peripheral arterial disease; BMI: body mass index; MPA: microscopic polyangiitis; GPA: granulomatosis with polyangiitis; EGPA: eosinophilic GPA; MPO: myeloperoxidase; P: perinuclear; PR3: proteinase 3; C: cytoplasmic; BVAS: Birmingham vasculitis activity score; FFS: five-factor score; ESR: erythrocyte sedimentation rate; CRP: C-reactive protein; PVR: pulse volume recording; ABI: ankle–brachial index; TcpO2: transcutaneous oxygen pressure; SPP: skin perfusion pressure.

**Table 2:** Concordance between PVR/ABI and TcpO2 or SPP in patients with AAV.

**Patients with AAV with both PVR/ABI and TcpO2 results (*n* = 16)**		
	Normal PVR/ABI	Abnormal PVR/ABI
Normal TcpO2	9 (56.3)	1 (6.3)
Abnormal TcpO2	6 (37.5)	0 (0)
**Patients with AAV with both PVR and SPP results (*n* = 23)**		
	Normal PVR/ABI	Abnormal PVR/ABI
Normal SPP	15 (65.2)	2 (8.7)
Abnormal SPP	5 (21.7)	1 (4.4)

Values are expressed as *n* (%). PVR: pulse volume recording; ABI: ankle–brachial index; TcpO2: transcutaneous oxygen pressure; SPP: skin perfusion pressure; AAV: ANCA-associated vasculitis; ANCA: antineutrophil cytoplasmic antibody.

**Table 3:** Systemic manifestations based on the items of the BVAS at PAD evaluation and poor outcomes during follow-up in patients with AAV (*n* = 54).

Variable	Value
At PAD evaluation	
Systemic manifestations (*n*, (%))	
General manifestation	21 (38.9)
Cutaneous manifestation	11 (20.4)
Mucous/Ocular manifestation	2 (3.7)
Otorhinolaryngological manifestation	24 (44.4)
Pulmonary manifestation	36 (66.7)
Cardiovascular manifestation	2 (3.7)
Gastrointestinal manifestation	1 (1.9)
Renal manifestation	33 (61.1)
Nervous systemic manifestation	28 (51.9)
During follow-up	
Poor outcomes	
ACM	5 (9.3)
ESKD	2 (3.7)
CVA	10 (18.5)
ACS	4 (7.4)

Values are expressed as *n* (%). BVAS: Birmingham vasculitis activity score; PAD: peripheral arterial disease; AAV: ANCA-associated vasculitis; ANCA: antineutrophil cytoplasmic antibody; ACM: all-cause mortality; ESKD: end-stage kidney disease; CVA: cerebrovascular accident; ACS: acute coronary syndrome.

**Table 4:** Comparison of systemic manifestation of BVAS items at PAD evaluation and poor outcomes during follow-up according to abnormal PVR/ABI, TcpO2, or SPP in patients with AAV.

	PVR/ABI (*n* = 54)			TcpO2 (*n* = 16)			SPP (*n* = 23)		
	Normal PVR/ABI (*n* = 51)	Abnormal PVR/ABI (*n* = 3)	*p* Value	Normal TcpO2 (*n* = 10)	Abnormal TcpO2 (*n* = 6)	*p* Value	Normal SPP (*n* = 17)	Abnormal SPP (*n* = 6)	*p* Value
Systemic manifestations based on BVAS									
General	20 (39.2)	1 (33.3)	1.000	2 (20.0)	4 (66.7)	0.118	4 (23.5)	2 (33.3)	0.632
Cutaneous	10 (19.6)	1 (33.3)	0.502	0 (0)	2 (33.3)	0.125	1 (5.9)	5 (83.3)	0.001
Mucous/ocular	2 (3.9)	0 (0)	1.000	0 (0)	0 (0)	N/A	1 (5.9)	0 (0)	1.000
Otorhinolaryngological	23 (45.1)	1 (33.3)	1.000	5 (50.0)	1 (16.7)	0.307	6 (35.3)	1 (16.7)	0.621
Pulmonary	35 (68.6)	1 (33.3)	0.255	9 (90.0)	5 (83.3)	1.000	14 (82.4)	3 (50.0)	0.279
Cardiovascular	2 (3.9)	0 (0)	1.000	1 (10.0)	0 (0)	1.000	1 (5.9)	0 (0)	1.000
Gastrointestinal	1 (2.0)	0 (0)	1.000	0 (0)	0 (0)	N/A	0 (0)	0 (0)	N/A
Renal	32 (62.7)	1 (33.3)	0.553	3 (30.0)	4 (66.7)	0.302	7 (41.2)	6 (100)	0.012
Nervous systemic	27 (52.9)	1 (33.3)	0.604	6 (60.0)	3 (50.0)	1.000	11 (64.7)	2 (33.3)	0.341
Poor outcomes									
ACM	4 (7.8)	1 (33.3)	0.257	1 (10.0)	0 (0)	1.000	1 (5.9)	1 (16.7)	0.462
ESKD	1 (2.0)	1 (33.3)	0.109	0 (0)	0 (0)	N/A	0 (0)	1 (16.7)	0.261
CVA	10 (19.6)	0 (0)	1.000	0 (0)	0 (0)	N/A	2 (11.8)	0 (0)	1.000
ACS	4 (7.8)	0 (0)	1.000	0 (0)	0 (0)	N/A	0 (0)	0 (0)	N/A

Values are expressed as *n* (%). BVAS: Birmingham vasculitis activity score; PAD: peripheral arterial disease; PVR: pulse volume recording; ABI: ankle–brachial index; TcpO2: transcutaneous oxygen pressure; SPP: skin perfusion pressure; AAV: ANCA-associated vasculitis; ANCA: antineutrophil cytoplasmic antibody; N/A: not applicable; ACM: all-cause mortality; ESKD: end-stage kidney disease; CVA: cerebrovascular accident; ACS: acute coronary syndrome.

## Data Availability

The dataset collected and/or analysed in the present study are avail-able on request from the corresponding author.

## Supplementary Materials

The following supporting information can be downloaded at: https://www.mdpi.com/article/10.3390/medicina61061074/s1, Figure S1: Follow-up duration based on each poor outcome. Figure S2: Clinical meanings of abnormal SPP.
