# Supplementary figures and images for: Clinical Significance of Peripheral Arterial Disease Evaluation in Patients with Antineutrophil Cytoplasmic Antibody-Associated Vasculitis
Source: Medicina (Kaunas). 2025 Jun 11;61(6):1074. doi: 10.3390/medicina61061074 (PMC12195056; doi:10.3390/medicina61061074)

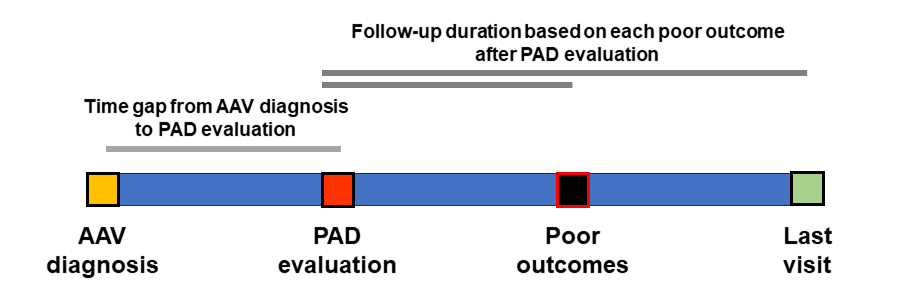

Supplement: Supplementary file 1 [file medicina-61-01074-s001.zip › SUPPLEMENTARY FIGURE1(PAD&AAV).tif]

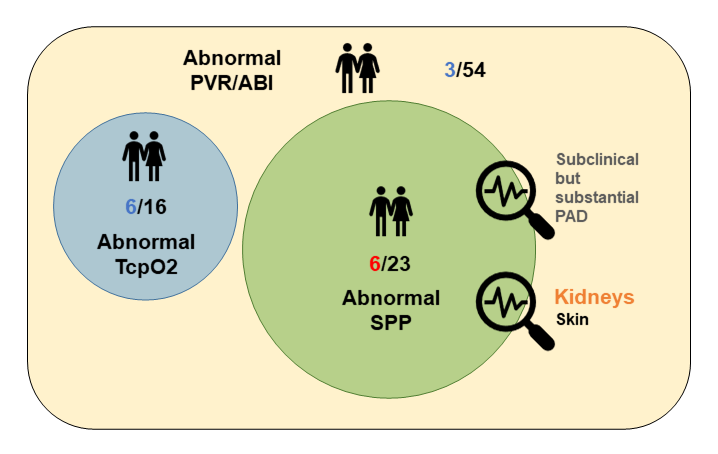

Supplement: Supplementary file 1 [file medicina-61-01074-s001.zip › SUPPLEMENTARY FIGURE2(PAD&AAV).tif]
